# Co-designing care for multimorbidity: a systematic review

**DOI:** 10.1186/s12916-024-03263-9

**Published:** 2024-02-07

**Authors:** Jennifer Sumner, Celeste Wen Ting Ng, Kimberly Ee Lin Teo, Adena Li Tyin Peh, Yee Wei Lim

**Affiliations:** 1 Alexandra Hospital, National University Health System, Singapore, Singapore; 2 Yong Loo Lin School of Medicine, National University of Singapore, Singapore, Singapore

**Keywords:** Multimorbidity, Chronic disease, Co-design, Systematic review

## Abstract

**Background:**

The co-design of health care enables patient-centredness by partnering patients, clinicians and other stakeholders together to create services.

**Methods:**

We conducted a systematic review of co-designed health interventions for people living with multimorbidity and assessed (a) their effectiveness in improving health outcomes, (b) the co-design approaches used and (c) barriers and facilitators to the co-design process with people living with multimorbidity. We searched MEDLINE, EMBASE, CINAHL, Scopus and PsycINFO between 2000 and March 2022. Included experimental studies were quality assessed using the Cochrane risk of bias tool (ROB-2 and ROBINS-I).

**Results:**

We screened 14,376 reports, with 13 reports meeting the eligibility criteria. Two reported health and well-being outcomes: one randomised clinical trial (*n* = 134) and one controlled cohort (*n* = 1933). Outcome measures included quality of life, self-efficacy, well-being, anxiety, depression, functional status, healthcare utilisation and mortality. Outcomes favouring the co-design interventions compared to control were minimal, with only 4 of 17 outcomes considered beneficial. Co-design approaches included needs assessment/ideation (12 of 13), prototype (11 of 13), pilot testing (5 of 13) (i.e. focus on usability) and health and well-being evaluations (2 of 13). Common challenges to the co-design process include poor stakeholder interest, passive participation, power imbalances and a lack of representativeness in the design group. Enablers include flexibility in approach, smaller group work, advocating for stakeholders’ views and commitment to the process or decisions made.

**Conclusions:**

In this systematic review of co-design health interventions, we found that few projects assessed health and well-being outcomes, and the observed health and well-being benefits were minimal. The intensity and variability in the co-design approaches were substantial, and challenges were evident. Co-design aided the design of novel services and interventions for those with multimorbidity, improving their relevance, usability and acceptability. However, the clinical benefits of co-designed interventions for those with multimorbidity are unclear.

**Supplementary Information:**

The online version contains supplementary material available at 10.1186/s12916-024-03263-9.

## Background

There is increasing awareness that health services and healthcare institutions designed for acute conditions do not adequately serve patients with multiple long-term conditions, often termed multimorbidity [1]. Consequently, many healthcare systems have adopted integrative care models for greater continuity and care coordination. Integrative care models prioritise patient-centric care and shift from a specialist-led mindset to a generalist-led care approach. The goal is that clinicians provide more holistic care than condition-specific care, with the hope that patients are empowered to understand and actively self-manage their conditions.

Despite the shift to integrative care, evidence on the effectiveness of interventions for those with multimorbidity is limited. In recent systematic reviews, inconsistent findings were found for organisational change (i.e. case management) and patient-level interventions (i.e. self-management support) for multimorbidity or comorbidity [2, 3]. Interventions that targeted common risk factors or functional difficulties appeared most promising, but more research is needed to confirm these results. Notably, the review by Smith et al. [3] also emphasises the importance of intervention design informed by stakeholder perspectives, for example, through participatory design methodologies [4].

Co-design, a participatory design methodology, is an increasingly common approach that facilitates the design of patient-centred services [4]. In general, co-design in healthcare involves active partnerships between patients, families, caregivers and care providers (among other stakeholders) to design a product together [5, 6]. The co-design process is typically iterative, involving multiple rounds of development and evaluation before reaching an outcome. By adopting a user-centred approach, co-design should ensure that healthcare interventions align with the needs, preferences and values of the people they aim to serve.

There are numerous examples of co-designed healthcare interventions, including self-management strategies, decision support systems and entire care models [7–9]. Studies suggest that co-designed interventions in healthcare improve outcomes, for example, increased patient satisfaction, improved care processes and safety, reduced medical errors, improved patient knowledge, enhanced service delivery and cost savings [10–14]. However, despite the popularity of co-design, the quality of evidence is relatively poor [15].

This review aimed to assess the impact of co-designed interventions for patients with multimorbidity and understand the experiences of co-design. Accordingly, we sought studies of co-designed health interventions for patients with multimorbidity which assessed (a) their effectiveness in improving health outcomes, (b) the approaches used and (c) what barriers and facilitators to the co-design process with people living with multimorbidity.

## Methods

This systematic review was conducted in accordance with the Preferred Reporting Items for Systematic Reviews and Meta-Analyses (PRISMA) and was registered on the PROSPERO database (ID: CRD42022330172). The PRISMA checklist can be found in Additional file 1. We looked to identify projects developing and testing a co-designed intervention targeting people living with multimorbidity or comorbidity. From these projects, data on health outcomes, the design approach and barriers and facilitators to the design process were extracted, if reported.

### Multimorbidity and comorbidity

Multimorbidity is a broad term defined as the coexistence of two or more chronic conditions [16]. A related term, comorbidity, refers to a patient with an index condition in combination with other condition(s) [16]. We included papers that considered both situations in this review.

### Search strategy

MEDLINE, EMBASE, CINAHL, Scopus and PsycINFO were searched from January 2000 up to 15 March 2022 using a combination of MeSH terms and keywords around the following themes: multimorbidity and co-design. We developed the search strategy with an information specialist. Additional file 2 contains the search strategy for MEDLINE.

### Inclusion and exclusion criteria

We considered all quantitative and qualitative studies, regardless of study design, according to the screening criteria in Table 1. Articles were excluded if they were not peer-reviewed, they were not published in English, or the article was not primary research.

**Table 1:** PICOS criteria

PICOS criterion	Definition
Population	The target population of the intervention is adults (≥ 18 years) with multimorbidity; defined as the existence of two or more chronic conditions. Studies on patients with comorbidities; defined as those with an index condition alongside other condition(s) were also eligible. Studies on older adults, where there was no explicit mention of multimorbidity, or comorbidity were excluded
Intervention	Any co-designed intervention for those with multimorbidity. We defined co-design as “the participation and equal collaboration between service providers, users, careers or the broad community to develop products or services which support health and well-being”. To be considered co-design, studies should demonstrate: 1) Multiple, iterative stages of development such as needs assessment, ideation, prototyping, pilot testing (i.e. usability) and impact evaluation 2) Evidence of collaboration between patients (or patient advocates), family or caregivers and healthcare providers AND 3) Evidence that patient (or patient advocates), family or caregiver involvement is for the development of a product or service for the benefit of multimorbid patients AND 4) Evidence that patients (or patient advocates), family, caregivers or healthcare providers are involved in the development process at more than one stage of the co-design process, i.e. meaningful contribution. Co-design stages include needs assessment, ideation, prototyping, pilot testing (i.e. usability) and impact evaluation
Comparison	For experimental studies (i.e. randomised controlled trials or prospective cohorts with a control group), the control group was considered to be those receiving a non-co-designed intervention or usual care
Outcomes	Any articles which included clinical or patient-reported outcome measure measures were eligible. Papers with data on the experience of the co-design process, including facilitators of and barriers to co-design, were also eligible
Study type	Any study design, including experimental or observational designs

### Study selection

Citations were downloaded and managed in EndNote X9. Seven researchers conducted an independent preliminary screening of titles and abstracts using the inclusion and exclusion criteria. To improve screening consistency amongst the researchers, the first hundred articles were screened by all. The group then met to discuss queries and align on screening disagreements. The process helped to refine the eligibility criteria and screening alignment. Studies with unclear eligibility were discussed as a group to reach a consensus. Studies that met the inclusion criteria underwent full-text screening by three researchers. Each article was independently dual-screened, and eligibility disagreements were resolved through discussion with a fourth researcher. Where studies were unclear, attempts were made to contact the main author to obtain more detailed information on the project.

### Data extraction

Four researchers performed the data extraction for the final sample of included studies. A second researcher checked the data extraction accuracy, and discrepancies were discussed and resolved. Extracted data items include study and population characteristics, intervention details, information on the co-design process, facilitators of and barriers to the co-design process and health and well-being outcome measures (e.g. clinical outcomes, health-related quality of life). The extraction sheet was piloted on two papers and refined before full data extraction. This helped the team to understand whether data items could be extracted and in what format, and whether there was additional relevant data the team should consider.

### Quality assessment

We used the updated Cochrane risk of bias tool (ROB-2) for randomised controlled trials (RCTs) and ROBINS-I for non-randomised studies [17, 18]. ROB-2 rates the risk of bias arising from the randomisation process, deviations from the intended intervention, missing outcome data, measurement of the outcome and selective reporting. Signalling questions are used to establish bias within each domain. ROBINS-I rates the risk of bias according to seven domains: confounding, selection bias, classification of interventions, deviation from intended intervention, missing data, outcome measurement and selected reporting. One researcher independently assessed the risk of bias; a second reviewer quality-checked the assessment and any disagreements were discussed until consensus.

### Data synthesis

Health and well-being outcomes from RCTs and controlled non-randomised studies were tabulated. Co-design approaches are discussed narratively, and facilitators and barriers to the co-design process were extracted and organised according to the co-design framework outlined by Pirinen [19]. The framework organises the facilitators and barriers of co-design into five domains: collaboration, origination, processes, implementation and methods.

## Results

**Fig. 1:**
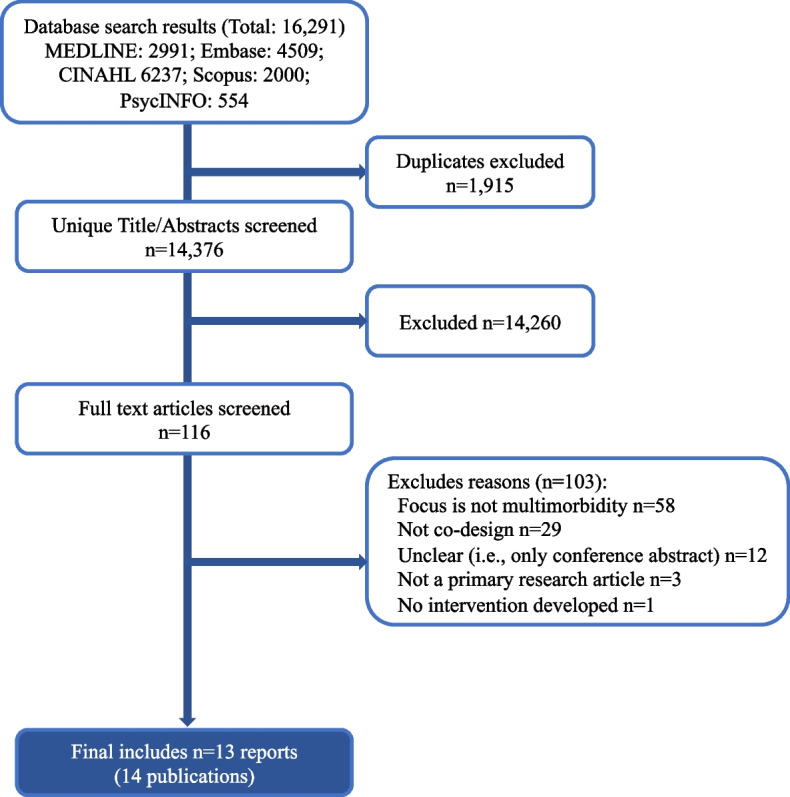
PRISMA flow diagram

We identified 16,291 reports, including 1915 duplicates, which we removed. Independent screening of the remaining 14,376 reports led to a further exclusion of 14,260 reports. Following the full-text screening, we excluded 100 reports leaving 13 reports included (Fig. 1). The most common reasons for exclusion were due to projects targeting single conditions or problems [20, 21], or using a non-co-design methodology to develop an intervention. In several cases, the intervention development process was not reported [22]. In others, a co-design approach was reported, but people living with multimorbidity were not involved in the design process [23], or were only engaged to understand the issue (needs assessment), demonstrating no evidence of sustained stakeholder involvement [24]. Finally, some studies were early in the development process, proposing future co-design work that had yet to be undertaken [25].

### Study characteristics

Table 2 presents the characteristics of the 13 included projects. Projects were from Europe (*n* = 9), Australia (*n* = 2), Canada (*n* = 1) and the USA (*n* = 1). Eight projects targeted adults with multimorbidity, the remainder targeted a principal chronic condition in combination with other chronic conditions. Principal conditions included stroke (*n* = 2), mental illness (*n* = 1), chronic obstructive pulmonary disease (*n* = 1) and diabetes (*n* = 1).

**Table 2:** Study characteristics

**Author, year, country**	**Aim of the project**	**Target population**	**Stages of co-design conducted**	**Stakeholders involved**	**Intervention designed**	**Final study conclusions**
Davis et al. 2020, USA [26]	To design a telehealth intervention programme for a rural population with multiple chronic conditions through an iterative process	Multimorbidity, unspecified conditions	• Needs assessment (*n* = N/R) • Ideation (*n* = N/R) • Prototype (*n* = N/R)	Telehealth experts, patient advocates, health policy faculty members, programme managers, medical directors, and healthcare professionals	A complex intervention based on the Model for Developing Complex Interventions in Nursing for patients with long-term conditions to minimise re-institutionalisation by using synchronous and asynchronous telehealth approaches to promote intervention effectiveness: remote autonomous monitoring, remote nursing assessment and treatment and care coordination	The Model for Developing Complex Interventions in Nursing provided a simple, structured process for designing a multifaceted telehealth intervention to minimise re-institutionalisation of participants with multiple chronic conditions
Easton et al. 2019, UK [27]	To develop a platform to support self-management for patients with an exemplary long-term condition (LTC; chronic pulmonary obstructive disease [COPD])	COPD with mental health conditions	• Needs assessment (*n* = 11) • Ideation (*n* = 19) • Prototype (*n* = 8)	Patients with COPD and healthcare professionals	An autonomous virtual agent supporting self-management for patients with COPD and potential mental health comorbidities using artificial intelligence during four scenarios: at the time of diagnosis, during acute exacerbations, during periods of low mood, and for general self-management	Supported self-management delivered via an autonomous virtual agent was acceptable to the participants. A co-design process has allowed the research team to identify key design principles, content, and functionality to underpin an autonomous agent for delivering self-management support to older adults living with COPD and potentially other LTCs
Ekstedt et al. 2021, Sweden [28]	To develop a user-centred design approach to identify and address the needs of older adults and healthcare professionals in the collaborative management of multiple chronic conditions,	Multimorbidity, unspecified conditions	• Needs assessment (*n* = 23) • Ideation (*n* = 10) • Prototype (*n* = 17) • Pilot (*n* = 7)	Patients with heart failure, COPD, or diabetes, carers, healthcare professionals, managers, administrators, quality developers, researchers and service designers	An e-health web-based application—ePATH (electronic Patient Activation in Treatment at Home)—with separate user interfaces for patients and healthcare professionals catering to the four essential pillars of self-management support	The feasibility study highlighted the importance of adequately addressing not only varying user needs but also the complex nature of healthcare organisations when implementing new services and processes in chronic care management
Gagnon et al. 2020, Canada [29]	To design and develop a user-centred patient portal for chronic disease management in primary care	Multimorbidity, unspecified conditions	• Ideation (*n* = N/R) • Prototype (*n* = 19) • Pilot (*n* = 18)	Patients with chronic illnesses, carers, healthcare professionals, designers, IT developers, and researchers	A patient portal (CONCERTO +) to promote patient engagement by improving patient care experiences, including personalising follow-ups, health education and communication between patients, caregivers and primary healthcare providers	Users generally found CONCERTO + intuitive and easy to navigate. Chronic patients and their informal caregivers are willing to use CONCERTO + to communicate with their primary healthcare team
Heim et al. 2016, Netherlands [30]	To report on the development, implementation and evaluation of a regional transitional care programme, aimed at improving the recovery rate of frail hospitalised older patients	Frailty with multimorbidity	• Needs assessment (*n* = N/R) • Ideation (*n* = N/R) • Real-world health and well-being evaluation *n* = 1933 (3-month follow-up)	Healthcare providers, older adults and knowledge institutes	A transitional care programme that improves integrated care for frail older patients reduces the risk of adverse outcomes after hospitalisation but has little effect on long-term care costs	By involving stakeholders in designing and developing the transitional care programme, the commitment of healthcare providers was secured and led to the development of an innovative and feasible programme. The collective improvement of integrated care for frail older patients reduced the risk for adverse outcomes after hospitalisation but has little impact on long-term care expenses
Horrell 2017, UK [31]	Through an iterative development process, create a patient-centred coordinated care (P3C) tool based on principles of promoting person-centred relationships with service users and practitioners	Multimorbidity, unspecified conditions	• Needs assessment (*n* = N/R) • Ideation (*n* = N/R) • Prototype (*n* = N/R) • Pilot (*n* = N/R)	Patients, carers, healthcare professionals, commissioners, and policy-makers	An organisational change tool (P3C-OCT) for assessing and monitoring an organisation’s and practitioners’ ability to provide personalised and coordinated care for people with multimorbidity, based on the principles of promoting person-centred relationships	The P3C-OCT provides a coherent approach to monitoring progress and supporting practice development towards P3C. It can be used to generate a shared understanding of the core domains of P3C at a service delivery level, and support re- organisation of care for those with complex needs. The tool can reliably detect change over time, as demonstrated in a sample of 40 UK general practices
Jinks et al. 2015, UK [32–34]	To develop and test the feasibility and acceptability of a practice nurse-led “enhanced” review for identifying, assessing and supporting management of joint pain, anxiety, and/or depression in patients attending routine long-term care reviews	Multimorbidity, unspecified conditions	• Needs assessment (*n* = N/R) • Ideation (*n* = N/R) • Prototype (*n* = N/R) • Real-world health and well-being evaluation	Patient and practice nurse advisory groups, practice nurses	A co-designed intervention of integrating joint pain and anxiety and/or depression into long-term care reviews by comprising tools for case-finding and initial patient assessment, evidence-based treatment options and signposting options to other services	The approach enabled the co-design of a new complex intervention of integrating joint pain and anxiety and/or depression into long-term care reviews in primary care consultations, and identification of training needs
Knowles et al. 2018, UK [12]	To generate interventions addressing safety issues for multimorbid patients in primary care	Multimorbidity, unspecified conditions	• Needs assessment (*n* = 16) • Ideation (*n* = 16) • Prototype (*n* = 11)	Multimorbid patients, carers, primary healthcare professionals	Two proposed interventions to address safety issues for multimorbid patients in primary care. (1) Automatic reminders to support adherence to a medication schedule. (2) An enhanced review provided by a pharmacist, developed collaboratively with the patients, embedded within the patients’ practice	The study demonstrates the value of bringing patients and professionals together to directly contribute to co-design
Lo et al. 2018, Australia [35]	To develop a new model of care for co-morbid diabetes and chronic kidney disease (CKD) by integrating healthcare at home, coordinating between primary and tertiary levels of care and promoting patient’s self-management and empowerment	Diabetes and CKD	• Needs assessment (*n* = 1279) • Ideation (*n* = not reported (N/R)) • Prototype (*n* = N/R)	Patients with diabetes and CKD, carers, healthcare professionals and consumer advocacy organisations—Diabetes and Kidney Health Australia	An integrated patient-centred model of care for multi-disciplinary care coordination	This model of care integrates with the patient-centred health-care home, allows coordination between primary and tertiary levels of care and promotes patient self-management and empowerment
Mehmet et al. 2020, Australia [36]	To analyse the role of social marketing using digital media initiatives to support the implementation of the Equally Well National Consensus Statement in rural and remote communities	Chronic physical and mental illness	• Needs assessment (*n* = 20) • Ideation (*n* = 45) • Pilot (*n* = N/R)	Patients with mental illness, carers, healthcare professionals, and service managers	A digital marketing strategy co-designed to help consumers, carers and clinicians to access quality, health-enhancing support and resources	The study proved that an embedded co-design process resulted in an integrated digital intervention mix that was useful in meeting the needs of rural stakeholders
Mercer et al. 2016, Scotland [37]	To develop and optimise a primary care-based complex intervention to enhance the quality of life of patients with multimorbidity in deprived areas	Multimorbidity, unspecified conditions	• Needs assessment (*n* = 32) • Ideation (*n* = 32) • Prototype (*n* = 32) • Pilot (*n* > 20) • Real-world health and well-being evaluation (*n* = 134)	Multimorbid patients, healthcare professionals, and representatives from third-sector organisations	The CARE Plus approach involves system changes, including longer consultations with relational continuity, patient–practitioner interaction changes using an empathic patient-centred structured approach, training and support for staff to deliver this and support for patient self-management	Enhancing primary care through a whole-system approach may be a cost-effective way to protect the quality of life for multimorbid patients in deprived areas
Porat et al. 2019, UK [38]	To design and evaluate an intervention informed by a learning health system approach to improve risk factor management and secondary prevention for stroke survivors with multimorbidity	Stroke with at least one other long-term condition	• Needs assessment (*n* = 45) • Ideation (*n* = 45) • Prototype (*n* = 44)	Stroke survivors, carers, healthcare professionals, commissioners, policymakers and researchers	A decision aid, DOTT (Deciding On Treatments Together) used in primary care during clinical consultations between the healthcare professional and stroke survivor, aims to facilitate shared decision-making on personalised treatments leading to improved treatment adherence and risk control	Adopting a user-centred data-driven design approach informed an intervention that is acceptable to users and has the potential to improve patient outcomes. Both stroke survivors and clinicians perceived the decision aid to be useful in consultations and eliciting preferences for treatment options
Sadler et al. 2017, UK [39]	To develop a process that engages stakeholders in the use of clinical and research data to co-produce potential solutions, informed by a Learning Health System, to improve long-term care for stroke survivors with multimorbidity	Stroke with at least one other long-term condition	• Needs assessment (*n* = 24) • Ideation (*n* = 45) • Prototype (*n* = 10)	Stroke survivors, family carers, healthcare professionals, service commissioners, policy-makers, service managers and researchers	A decision tool to identify stroke survivors at risk for a recurrent stroke, enhance shared decision-making between patients and clinicians and propose optical care pathways to reduce stroke survivor’s risk factors, improving secondary prevention after stroke	Stakeholder engagement to identify data-driven solutions is feasible but requires resources. Further work is required to evaluate the impact and implementation of data-driven interventions for long-term stroke survivors

Interventions had variable aims, such as improvement of a specific aspect of care, i.e. care coordination, communication, shared decision making or care transition. Other interventions supported self-management, were novel treatments or were designed to assess the quality-of-care delivery. Technology was also leveraged in some cases, including web portals, artificial intelligence and apps.

### Assessment of health and well-being outcomes

Out of the 13 projects included, two (an RCT *n* = 134 and cohort study *n* = 1933) reported on the health and well-being effects of the interventions [30, 37]. One further RCT only reported baseline data, so no outcome data could be extracted [32]. Outcome measures included quality of life, functional status, healthcare utilisation and mortality. In the RCT (Table 3) [37], only one out of eight measures favoured the intervention group (negative well-being subscale of the well-being scale-12). In the observational study [30], activities of daily living (relative risk (RR) and confidence interval (CI) 0.74 (0.58, 0.95)), healthcare utilisation (RR and CI 0.45 (0.29, 0.70)) and risk of adverse outcome (RR and CI 0.72 (0.60, 0.87)) (a composite of death, decline in activities of daily living function and high healthcare demand post admission) favoured the co-designed intervention in the frail cohort. No differences were found in the non-frail cohort.

**Table 3:** Health and well-being outcomes from Mercer et al. [37]

**12-month follow-up**	**Adjusted mean difference (confidence interval)** ***n* = 134**
EQ5D-5L	0.06 (− 0.02, 0.14)
W-BQ12 General	1.99 (− 0.27, 4.24)
W-BQ12 Negative	− 1.30 (− 2.16, − 0.43)
W-BQ12 Energy	0.31 (− 0.55, 1.17)
W-BQ12 Positive	0.57 (− 0.56, 1.70)
HADS Depression	− 1.25 (− 2.53, 0.03)
HADS Anxiety	− 0.91 (− 1.93, 0.12)
Self-efficacy	0.07 (− 0.69, 0.83)
Self-esteem	0.74 (− 0.96, 2.45)

*EQ-5D *European quality of life index-5 dimensions, *WBQ12 *Well-being scale-12, *HADS *Hospital Anxiety and Depression Scale

### Risk of bias

Mercer et al. [37] were rated as having a low risk of bias in all domains except outcome measurement, which was rated as moderate. Heim et al. [30] were rated as having a high risk of confounding bias; a moderate risk of bias in the selection, outcome measurement and reporting domains; and a low risk of bias in all other domains.

### Co-design approaches used

Co-design stages included needs assessment/ideation (12 of 13), prototyping (11 of 13), pilot testing (5 of 13) (i.e. focus on usability) and health and well-being evaluations (3 of 13). All studies involved patients or patient advocates and healthcare professionals in their co-design process, and nine included carers. Less commonly included stakeholders were commissioners, researchers, service managers and policy-makers.

### Needs and ideation

For needs and ideation, a myriad of methods were used, including focus group discussions, interviews, literature reviews and expert panels. Less common techniques included clinical observations, questionnaires and a review of existing patient informational leaflets.

### Prototyping

Prototyping helped to gather feedback from stakeholders on the proposed solution. Prototypes ranged in sophistication, from simple pen and paper sketches to functional mock-ups. For example, in one study, telehealth vendors were invited to demonstrate their products so stakeholders could assess factors like functionality, ease of use and cost [26]. In another example, a prototype of an autonomous chatbot was tested with simulated interactions in a living lab (i.e. a mock real-world environment) to gauge the user experience and elicit feedback [27]. Most prototypes followed an iterative development process. For example, in one study, a decision support system underwent four prototyping testing and refinement rounds [38]. Prototypes were typically created to evaluate usability [29, 38], readability [30, 31] and content validity [31]. Prototyping also helped to facilitate discussion, which aided the design process and built knowledge among the stakeholders. Evaluation approaches included focus groups or interviews, expert panel reviews, or workshops with discussion.

### Pilot testing

Pilot evaluations were conducted in the real-world setting, primarily to test and refine the functionality of the intervention before assessing clinical effects. Pilot study outcomes were both subjective and objective. Examples of subjective outcomes included usability, acceptability and satisfaction [28, 29, 31, 36, 37]. Objective outcome examples included page views and download rates [36]. Some studies conducted more than one pilot evaluation and used several rounds to test and finesse their intervention [28].

### Barriers and facilitators of the co-design process

**Fig. 2:**
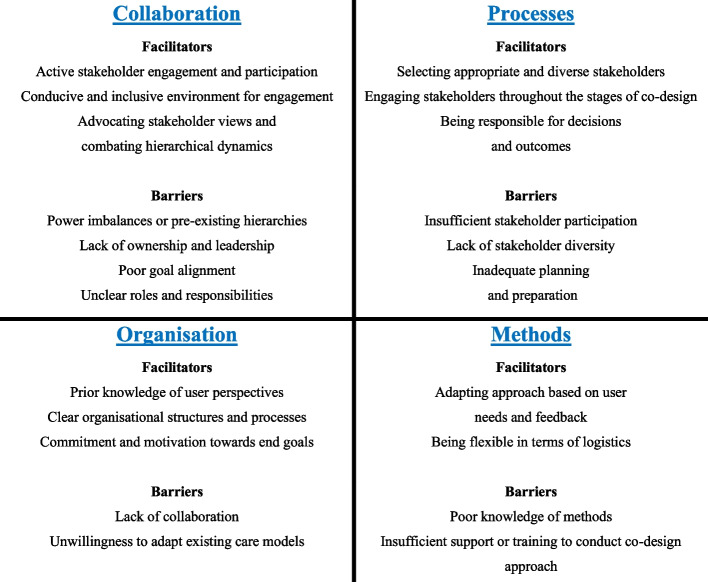
Facilitators and barriers of co-design identified in our included studies

Ten projects reported on the barriers and facilitators of the co-design process. Barriers and facilitators were categorised into four of the five domains outlined by Pirinen [19]. For the fifth domain ‘barriers to the implementation of co-designed solutions’, we identified no findings. Figure 2 provides a summary of the main factors impacting the co-design process.

The most common barrier to co-design is related to participant interactions. Examples included poor stakeholder interest or difficulty maintaining project momentum [28, 36, 39], passive participation [27], or power imbalances between participants [40]. Representativeness of the design group and, correspondingly, the appropriateness of the output was another frequently mentioned barrier to co-design [12, 26, 27, 40]. Other less common co-design barriers included inadequate skills and knowledge of the co-design approach, poor understanding of the problem or solution, logistical challenges (i.e. scheduling and time commitment) and managing conflicting feedback [26, 36].

Reported enablers of co-design most frequently related to the chosen co-design methods, such as being flexible in accommodating schedules and opting for smaller rather than larger group work to facilitate discussions [39, 40]. It was also considered important to establish a conducive environment where stakeholders would actively engage and feel comfortable expressing their views [40]. Often, this involved advocating for stakeholder views and combating group hierarchies to ensure that all voices are heard [27, 40]. Finally, commitment to the process and taking responsibility for decisions helped promote a sense of ownership among participants, facilitating the co-design process [28].

## Discussion

In this systematic review of 13 co-designed intervention studies with people living with multimorbidity, we found that only two reported health and well-being outcomes. Furthermore, the effects of the co-designed interventions were minimal; only 4 of 17 outcomes were considered beneficial compared to the control. The co-design development phases included needs assessment/ideation, prototyping, pilot testing (i.e. focus on usability) and health and well-being evaluations. However, not every project went through every phase of co-design. The most commonly reported challenges to the co-design process were related to participant interactions and the inability to engage a breadth of participants during design. Overall, the authors reported that the co-design approach aided in the design of novel services and interventions, improving their relevance, usability and acceptability. However, the clinical benefits of co-designed interventions are unclear.

We found variability in the co-design approaches undertaken in the included projects, such as in the stages of co-deign undertaken, the degree of stakeholder involvement and methodological techniques used during development processes. The lack of a single, uniform conceptualisation of co-design may explain this. It was common for the included projects to utilise different definitions of co-design, which impacts the approach and aim of co-design [41–45]. For example, terms such as ‘equal partnerships’ and ‘together in partnerships’ introduce considerable ambiguity, and inferences may differ. This variability in co-design nomenclature creates significant challenges in executing a genuine co-design approach. Accordingly, despite claiming to use a co-design approach, we excluded many studies for limited stakeholder involvement or minimal stakeholder interaction (i.e. partnership). Researchers can avoid adopting poor methodology by accessing reliable co-design resources to guide their study design [46–48].

We considered projects targeting both multimorbidity and comorbidity in our review. An intervention design, addressing those with multiple chronic conditions, presents unique challenges compared to a comorbid intervention design that targets an index condition alongside other conditions. While designing in the context of multimorbidity accounts for the interconnectedness of conditions, difficulties arise in defining and measuring outcomes that have made synthesising evidence and drawing conclusions not straightforward [3]. Efforts to address obstacles to evidence synthesis in multimorbidity research include the development of a core set of indicators for studies in this field [49]. In contrast, intervention design with a comorbid focus, which emphasises a patient’s needs related to an index condition, may be more straightforward but risks overlooking the holistic needs of patients with multiple conditions. Although we could not examine the distinct effects of interventions for those with multimorbidity and comorbidity in this review, as the co-design evidence base grows, this should be re-examined.

In our review, a significant portion of projects leveraged technology. Examples include apps [28, 50], online web portals [27, 29] and other digital media [36]. Those with multimorbidity tend to be older adults, and it is not uncommon for this group to be less accepting or less able to use technology [51]. Furthermore, designing and evaluating technology with older adults can also be difficult [52, 53]. The challenges of designing technology with older adults can be managed by adhering to inclusive design principles such as the Universal Design principles (equitable use and flexibility, perceptibility, tolerance for error, simplicity, low effort and accessibility) [54]. By involving older adults in the co-design process, designers can customise the technological interventions to facilitate adoption and improve ease of routine use.

In our included studies, we found numerous barriers related to the co-design processes. One significant barrier was the presence of pre-existing hierarchies, which hindered collaboration efforts in some projects. For instance, patients or non-professional groups often had less recognition or acknowledgement of their contribution, limiting their active involvement in the co-design process. Some projects found that participants had a poor understanding of co-design and the process involved, which hindered the ongoing work and outputs. Others reported that their projects may have limited generalisability due to a lack of diversity in the participants recruited [15]. For example, two projects struggled with adequate representation of healthcare professionals in their design group, risking the discussion being dominated by other stakeholders [12, 55].

### Limitations

Co-design is not defined consistently in the literature and includes a high degree of variability in terminology. Therefore, it is possible that we missed some relevant papers because we did not use all pertinent co-design terms in our literature searches. By extending our selection of search phrases, we attempted to minimise this risk. In addition, due to the lack of a standardised co-design definition, we a priori set criteria to define co-design, such as deciding that participants must be involved in at least two co-design processes. Others may have different interpretations. Thus, we may have excluded relevant articles. Furthermore, we did not include specific conditions in our search strategy, which may mean we missed eligible articles. Finally, we could not draw conclusions on the impact of the co-designed interventions on health and well-being outcomes due to the limited evidence identified. Multiple statistical comparisons within these studies also introduced bias, further complicating the interpretation of their results.

### Recommendations for co-design

First, clinicians and researchers engaging in co-design should recognise the complexity and diversity of people with multimorbidity. Patients’ conditions, symptoms, treatment regimens and challenges may vary significantly. Thus, everyone’s unique needs and circumstances need to be considered throughout when undertaking a co-design project. Furthermore, the heterogeneity of people living with multimorbidity requires careful consideration of what stakeholders need to be involved. To ensure inclusivity and comprehensive insights, researchers should strive to involve a wide range of individuals and groups. Second, stakeholder interactions must be managed. Being flexible and using a variety of engagement approaches can help facilitate the encounters between stakeholders. In cases of power imbalance among stakeholders, design teams must advocate for fair representation to ensure that perspectives from all stakeholders are captured. Third, while many co-design guidelines exist [56–58], the concept of co-design remains heterogeneous, with no unified guide on reporting or evaluating such studies. There is a need for better standardisation in reporting. The COcreation REsearch Standards (CORES) project is underway to improve reporting standards, and findings will be published on the Equator Network website in the future [59].

### Recommendations for multimorbid and comorbid research

Current opinion suggests that an RCT design may be unsuitable for evaluating interventions for those with multimorbidity [2, 3, 60]. Future work should consider pragmatic research designs, which can more adeptly consider intervention complexities and the diversity in people living with multimorbidity. Longitudinal work is also lacking; studies gauging intervention impact over time should be prioritised, in addition to implementation evaluations, to understand real-world dynamics and what works best for whom. Finally, core indicators such as those developed by Smith et al. as part of the Core Outcomes Measures in Effectiveness Trials initiative must be included in studies to facilitate evidence synthesis and policy decisions [49].

## Conclusions

Co-design is a participatory design approach that is becoming more prevalent in healthcare to improve services. However, the benefits of co-designed interventions for people with multimorbidity remain unclear. Future efforts should continue to involve stakeholders in healthcare redesign but should also commit to evaluating the impact of co-design interventions. More significant consideration of mental health and specific disease combinations is also needed to account for the complexities of care for those with multimorbidity.

### Supplementary Information

**Additional file 1. **PRISMA 2020 Checklist.

**Additional file 2.** MEDLINE search strategy.

## Data Availability

Not applicable.

## Abbreviations

CI: Confidence interval
CKD: Chronic kidney disease
COPD: Chronic obstructive pulmonary disease
CORES: The cocreation research standards
EQ-5D: European Quality of Life index-5 dimensions
HADs: Hospital anxiety and depression scale
LTC: Long-term condition
NR: Not reported
P3C: Patient-centred coordinated care
PRISMA: Preferred reporting items for systematic reviews and meta-analyses
RCTs: Randomised controlled trials
ROB-2: Risk of Bias Tool-2
ROBINS-I: Risk Of bias in non-randomised studies of interventions
RR: Relative risk
WBQ12: Well-being scale-12
