# Supplementary material for: Co-designing care for multimorbidity: a systematic review
Source: BMC Med. 2024 Feb 7;22:58. doi: 10.1186/s12916-024-03263-9 (PMC10848537; doi:10.1186/s12916-024-03263-9)
Supplement: Supplementary file 2 — Additional file 2. MEDLINE search strategy. [file 12916_2024_3263_MOESM2_ESM.docx]

**Co-designing multimorbid care: A systematic review**

Supplement 2: Medline search strategy

1. multimorbid* OR multi‐morbid*:ab,ti
2. 'complex needs':ab,ti
3. 'multiple long-term conditions':ab,ti
4. 'coexisting conditions':ab,ti
5. multidisease* OR multi‐disease*:ab,ti
6. ((concomit* OR concurren* OR multi* OR multiple) adj3 (ill* OR condition* OR morbidit* OR syndrom* OR disorder* OR disease*)):ab,ti
7. (chronic* adj3 (disease? OR ill* OR care OR condition? OR disorder* OR health* OR medication* OR syndrom* OR symptom*) adj3 (manag* OR treatment* OR intervention* OR program*)):ab,ti
8. comorbidity OR 'multiple chronic conditions':ab,ti
9. ((comorbid* OR co‐morbid*) adj3 (intervention* OR treatment*)):ab,ti
10. multipatholog* OR multi?patholog*:ab,ti
11. polypatholog* OR poly?patholog*:ab,ti
12. pluripatholog*:ab,ti
13. polydiagnos*:ab,ti
14. multicondition*:ab,ti
15. multidisor*:ab,ti
16. patient-centered care/
17. ((patient OR user OR consumer OR stakeholder) adj1 (centered OR centred OR centric OR participa* OR activat* OR engag* OR empower* OR involve* OR voice OR view OR perspective OR satisfied OR satisfactory OR satisfaction OR experience* OR advoca* OR behav* OR evaluate OR evaluated OR evaluation OR communicat* OR collaborat*)):ab,ti
18. patient satisfaction/
19. patient AND 'public involve*':ab,ti
20. patient participation/
21. ((shared OR joint) adj2 decision):ab,ti
22. (living adj1 (lab OR laboratory OR laboratories)):ab,ti
23. 'collective creativity':ab,ti
24. (empathetic* adj2 design*):ab,ti
25. 'value sensitive design*':ab,ti
26. 'human cent* design*':ab,ti
27. 'cultur* chang*':ab,ti
28. 'consumer behavio?r':ab,ti
29. 'consumer-directed care':ab,ti
30. 'inclusive design*':ab,ti
31. (citizen* adj1 (jury OR juries)):ab,ti
32. (health adj2 consumer):ab,ti
33. (action adj2 research):ab,ti
34. 'lived experience':ab,ti
35. 'open innovation':ab,ti
36. 'collective intelligence':ab,ti
37. 'community participation':ab,ti
38. (community adj1 participa*):ab,ti
39. (consumer adj1 (drive OR driven)):ab,ti
40. 'experience based design*':ab,ti
41. 'participa* ergonomics':ab,ti
42. 'participa* design*':ab,ti
43. 'persona based design*':ab,ti
44. 'co produce' OR 'co producing' OR 'co production' OR coproduc*:ab,ti
45. 'co creat*' OR cocreat*:ab,ti
46. 'co design*' OR codesign*:ab,ti
47. 'co care' OR 'co caring' OR 'co cared' OR cocar*:ab,ti
48. 'co commiss*' OR cocommiss*:ab,ti
49. 'co decide' OR 'co decision' OR 'co decided' OR codeci*:ab,ti
50. 'co deliver*' OR codeliver*:ab,ti
51. 'co evaluat*' OR coevaluat*:ab,ti
52. 'co implement*' OR coimplement*:ab,ti
53. 'co construct*' OR coconstruct*:ab,ti
54. 'co innovat*' OR coinnovat*:ab,ti
55. 'co learn*' OR colearn*:ab,ti
